# Supplementary material for: The early life microbiota protects neonatal mice from pathological small intestinal epithelial cell shedding
Source: FASEB J. 2020 Apr 6;34(5):7075–88. doi: 10.1096/fj.202000042R (PMC7610993; doi:10.1096/fj.202000042R)
Supplement: Supplementary file 1 — Supplementary Material [file FSB2-34-7075-s001.pdf]

	A	B	C	D	E	F	G	H	I	J	K	L
1	Pos Cntrl	Pos Cntrl	Neg Cntrl	Neg Cntrl	BLANK	BLC	CD30 L	Eotaxin	Eotaxin-2	Fas Ligand	Fractalkine	GCSF
2	Pos Cntrl	Pos Cntrl	Neg Cntrl	Neg Cntrl	BLANK	BLC	CD30 L	Eotaxin	Eotaxin-2	Fas Ligand	Fractalkine	GCSF
3	GM-CSF	GM-CSF	IL-1α	IL-1β	IL-2	IL-7	IL-4	IL-6	IL-9	IL-10	IL-12 p40/p70	IL-12 p70
4	GM-CSF	GM-CSF	IL-1α	IL-1β	IL-2	IL-7	IL-4	IL-6	IL-9	IL-10	IL-12 p40/p70	IL-12 p70
5	IL-13	IFN-γ	I-TAC	KC	Leptin	LIX	Lymphotactin	MCP-1	MCSF	MIG	MIP-1α	MIP-1γ
6	IL-13	IFN-γ	I-TAC	KC	Leptin	LIX	Lymphotactin	MCP-1	MCSF	MIG	MIP-1α	MIP-1γ
7	RANTES	SDF-1	TCA-3	TECK	TIMP-1	TIMP-2	TNF-α	sTNF RI	sTNF RII	BLANK	BLANK	Pos Cntrl
8	RANTES	SFD-1	TCA-3	TECK	TIMP-1	TIMP-2	TNF-α	sTNF RI	sTNF RII	BLANK	BLANK	Pos Cntrl

Supplementary Table 1: Antibody array layout

A

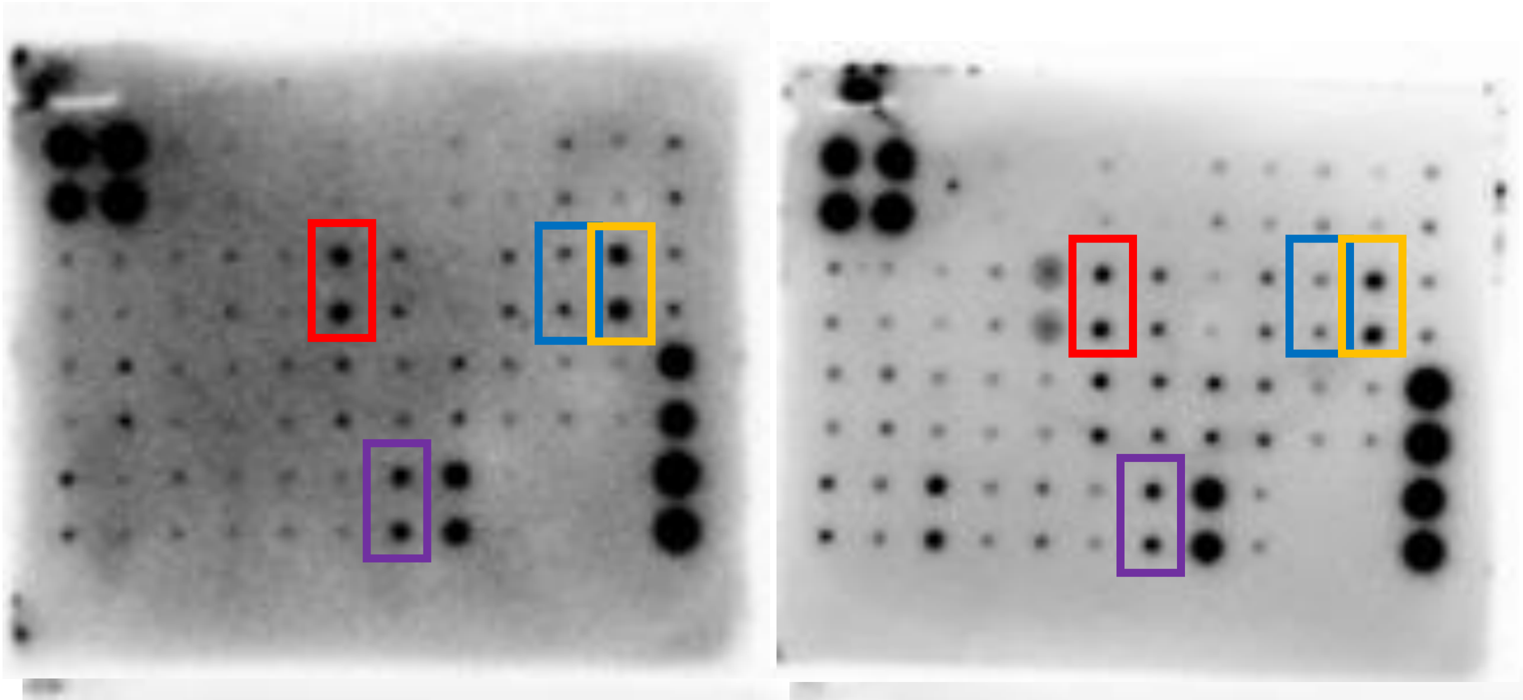

B

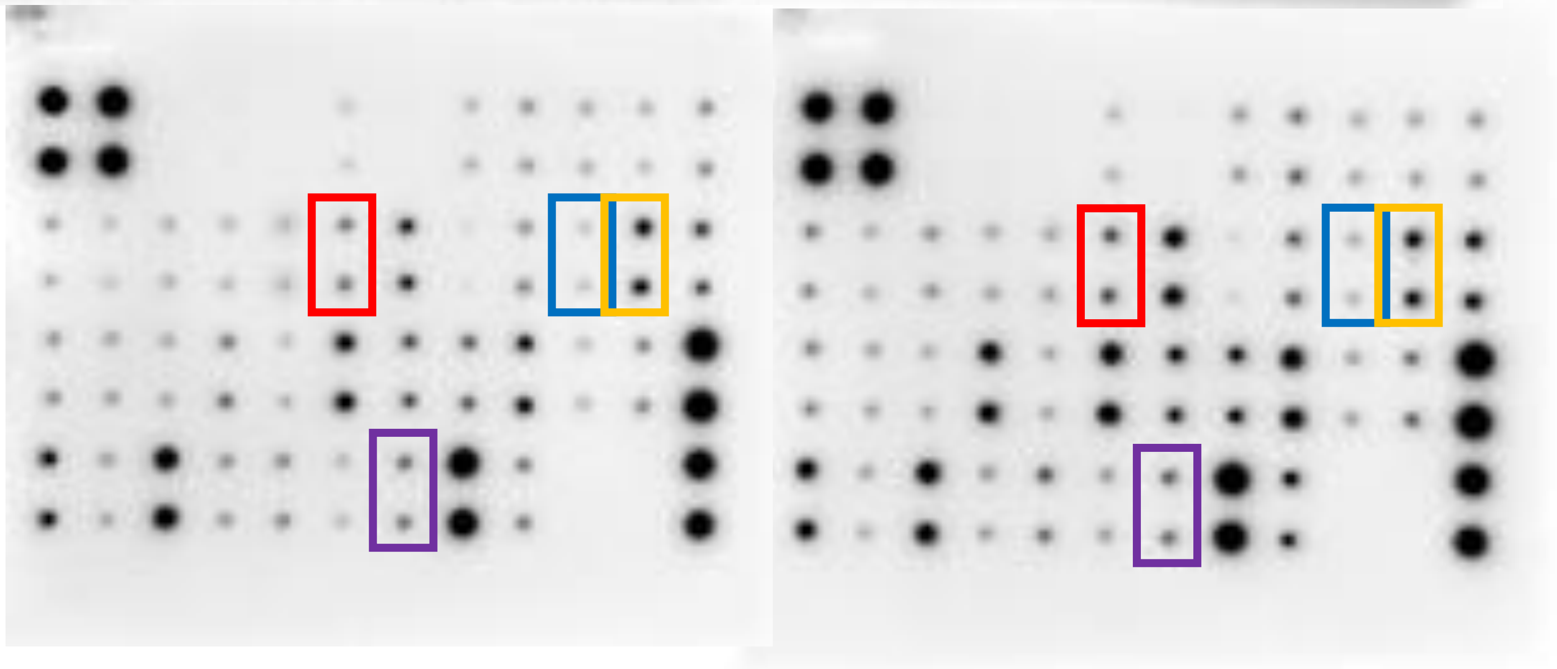

Supplementary Figure 1: Neonatal mice have altered cytokine responses
Antibody arrays were used to assess cytokine changes in neonatal versus adult homogenised small intestinal samples (see **Supplementary Table 1** for full play layout). (**A**) Two experimental replicate antibody arrays from D14 neonates after treatment with 1.25mg/kg LPS. (**B**) Adult (8-10 week old) antibody arrays after LPS treatment. Coloured boxes highlight elevated differences in neonatal homogenates vs. adult samples; red (IL-7), blue (IL-10), yellow (IL-12), and purple (TNF-α).

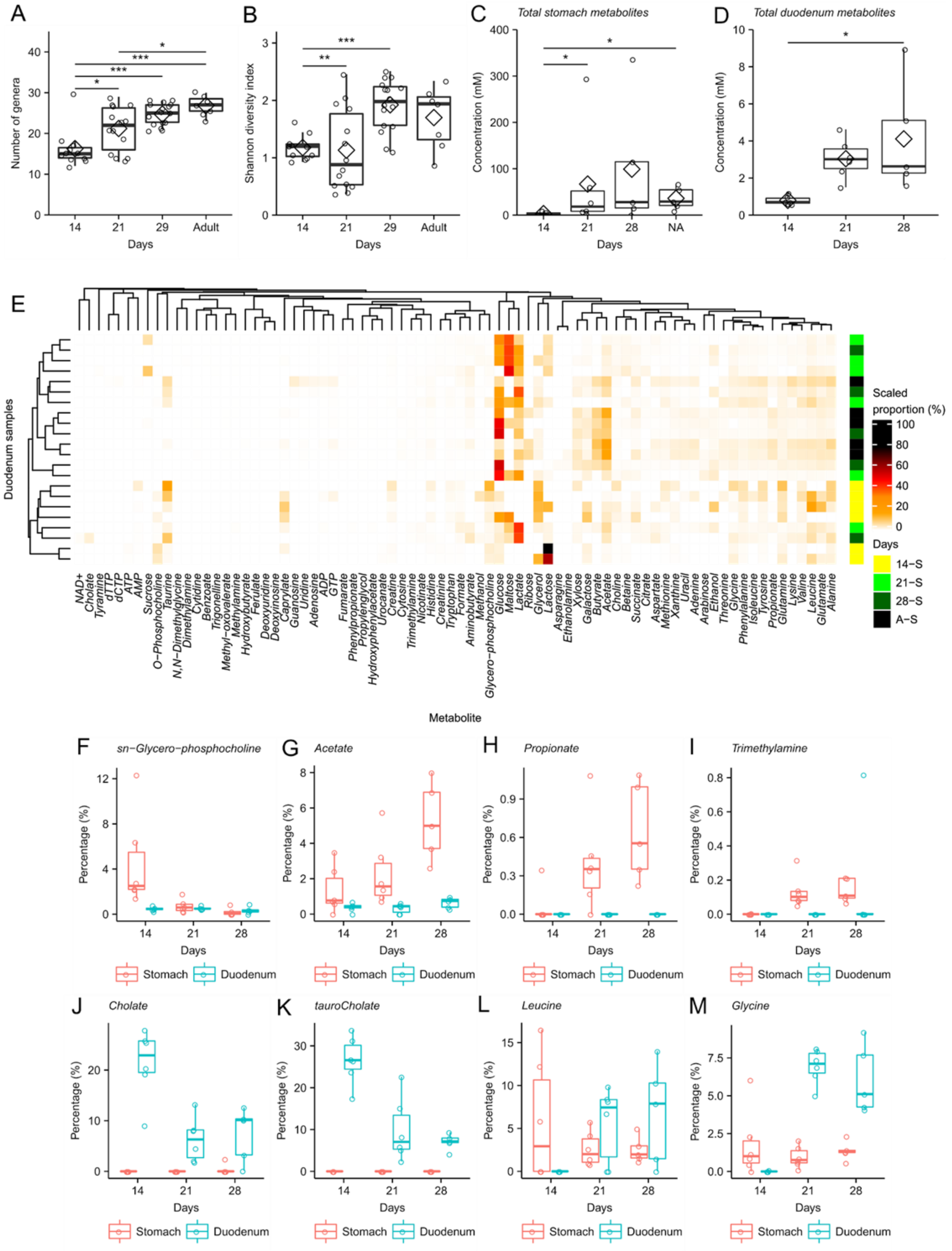

Supplementary Figure 2: Microbiota diversity, total metabolites and stomach metabolite composition. (**A**) Number of genera detected in each faecal sample. (**B**) The Shannon Diversity index of the microbiota in each sample at the genus level. (**C**) The total concentration of metabolites in stomach contents. (**D**) The total concentration of metabolites in duodenum contents. (**E**) Heatmap showing proportions of metabolites in stomach contents determined using NMR analysis and clustered using a Bray-Curtis matrix shows distinct grouping of metabolites at mice aged 14 days. (**F**) sn-Glycero-phosphocholine, (**G**) Acetate, (**H**) Propionate, (**I**) Trimethylamine, (**J**) Choline, (**K**) tauroCholine, **(L)** Leucine, and (**M**) Glycine. Boxplots show mean (diamond), median (solid bar), and interquartile range while circles show individual samples. Statistical significance determined by ANOVA with post hoc Tukey test or Kruskal–Wallis test with post hoc Pairwise Wilcoxon Tests. *= $p < 0.05$, **= $p < 0.01$, and ***= $p < 0.001$.
